# Exploring the Prevalence of Tinnitus and Ear-Related Symptoms in China After the COVID-19 Pandemic: Online Cross-Sectional Survey

**DOI:** 10.2196/54326

**Published:** 2024-04-24

**Authors:** Di Wang, Peifan Li, Xiaoling Huang, Yixuan Liu, Shihang Mao, Haoning Yin, Na Wang, Yan Luo, Shan Sun

**Affiliations:** 1 Research Service Office Eye & ENT Hospital Fudan University Shanghai China; 2 NHC Key Laboratory of Hearing Medicine Research Fudan University Shanghai China; 3 State Key Laboratory of Medical Neurobiology Fudan University Shanghai China; 4 Department of Otolaryngology Head and Neck Surgery Second Affiliated Hospital of Anhui Medical University Hefei China; 5 No.2 High School Of East China Normal University Shanghai China; 6 School of Public Health Fudan University Shanghai China; 7 Tinnitus Hyperacusis Center, Otolaryngology Research Institute Eye & ENT Hospital Fudan University Shanghai China; 8 Clinical Research Unit of the Eye & ENT Hospital Fudan University Shanghai China

**Keywords:** COVID-19 pandemic, tinnitus, ear-related symptoms, online survey, prevalence, ear-related, China, cross-sectional, complex, heterogeneous, symptom, symptoms, Chinese, population, investigate, health care, exploratory, teen, teens, teenager, teenagers, older adult, older adults, elder, elderly, older person, older people, COVID-19, regression analysis

## Abstract

**Background:**

Tinnitus is a complex and heterogeneous disease that has been identified as a common manifestation of COVID-19. To gain a comprehensive understanding of tinnitus symptoms in individuals following COVID-19 infection, we conducted an online survey called the China Ear Nose and Throat Symptom Survey in the COVID-19 Pandemic (CENTSS) among the Chinese population.

**Objective:**

Our objective was to investigate tinnitus and ear-related symptoms after COVID-19 infection in the Chinese population, with the aim of providing a solid empirical foundation for improved health care. The findings from CENTSS can contribute to the development of enhanced management strategies for tinnitus in the context of long COVID. By gaining a better understanding of the factors contributing to tinnitus in individuals with COVID-19, health care providers can tailor interventions to address the specific needs of affected patients. Furthermore, this study serves as a basis for research on the long-term consequences of COVID-19 infection and its associated tinnitus symptoms.

**Methods:**

A quantitative, online, cross-sectional survey study design was used to explore the impact of the COVID-19 pandemic on experiences with tinnitus in China. Data were collected through an online questionnaire designed to identify the presence of tinnitus and its impacts. Descriptive statistics were used to analyze individuals' demographic characteristics, COVID-19 infection–related ear symptoms, and the cognitive and emotional implications of tinnitus. Univariable and multivariable logistic regression analyses were used to model the cross-sectional baseline associations between demographic characteristics, noise exposure, educational level, health and lifestyle factors, and the occurrence of tinnitus.

**Results:**

Between December 19, 2022, and February 1, 2023, we obtained responses from 1262 Chinese participants representing 24 regions, with an average age of 37 years. Among them, 540 patients (42.8%) reported experiencing ear-related symptoms after COVID-19 infection. Only 114 (9%) of these patients sought medical attention specifically for their ear symptoms, while 426 (33.8%) did not seek hospital care. Tinnitus emerged as the most prevalent and impactful symptom among all ear-related symptoms experienced after COVID-19 infection. Of the respondents, female participants (688/888, 77.78%), younger individuals (<30 years), individuals with lower education levels, participants residing in western China, and those with a history of otolaryngology diseases were more likely to develop tinnitus following COVID-19 infection.

**Conclusions:**

In summary, tinnitus was identified as the most common ear-related symptom during COVID-19 infection. Individuals experiencing tinnitus after COVID-19 infection were found to have poorer cognitive and emotional well-being. Different ear-related symptoms in patients post–COVID-19 infection may suggest viral invasion of various parts of the ear. It is therefore crucial to monitor and manage hearing-related changes resulting from COVID-19 as clinical services resume.

## Introduction

Auditory-related conditions such as dizziness, tinnitus, and earache have been identified as common symptoms of COVID-19 [1]. These symptoms can vary in duration, ranging from acute (lasting up to 4 weeks) to ongoing (lasting 4-12 weeks) to lasting more than 12 weeks, which is commonly referred to as “long COVID” [2]. The National Institute for Health and Care Excellence (NICE) [3] has also recognized tinnitus, dizziness, and earache as common symptoms of long COVID. According to a systematic literature review, approximately 8% of individuals with COVID-19 reported experiencing tinnitus [4].

Tinnitus is characterized by the perception of sound in the absence of an external sound source [5], and individuals with tinnitus may experience buzzing, ringing, or hissing sounds. It is one of the most prevalent inner ear disorders worldwide, affecting 5% to 43% of adults [6]. Tinnitus often leads to irritation, anxiety, depression, and insomnia and can also impair concentration, negatively impacting quality of life [7]. In severe cases, tinnitus can even contribute to suicidal tendencies [8]. Additionally, because tinnitus frequently coexists with hearing loss, it can result in communication difficulties, particularly among older adults, leading to social isolation, cognitive impairment, and possibly dementia [9]. Given its significant impact on health and quality of life, it is crucial to develop methods for early diagnosis and treatment of tinnitus.

COVID-19 triggers an immune response that can cause damage to cells and tissues [10]. As a result, the virus causing COVID-19 has the potential to affect the cochlea, auditory nerve, and central nervous system [11]. Inflammatory responses induced by the virus are speculated to be a potential cause of tinnitus in patients with long COVID. Direct viral infection may also contribute to the development of tinnitus [12]. Moreover, COVID-19 has been associated with disruptions in daily routines and social isolation, which can lead to elevated levels of depression, anxiety, and perceived stress, potentially exacerbating the perception of tinnitus [13]. Understanding the impact of long COVID on the auditory system and psychological well-being is crucial, and it can provide insights into the characteristics of patients experiencing tinnitus after COVID-19 infection and can inform the development of effective treatment strategies.

Furthermore, in an effort to break the chain of transmission, numerous regional lockdowns were implemented between 2019 and 2022. China recently relaxed certain restrictions, including quarantine rules for travelers and mask-wearing mandates, in December 2022, despite maintaining a comprehensive zero-COVID policy. This study focused on the specific characteristics of ear-related symptoms, particularly tinnitus, that may arise following the lifting of lockdown measures in the context of mass COVID-19 infections. The potential for tinnitus to become more severe due to long COVID presents a concerning challenge, necessitating further research to determine the potential exacerbation of tinnitus following prolonged COVID-19.

To gain a better understanding of the characteristics of patients experiencing tinnitus symptoms following COVID-19 infection, we conducted an online survey called the China Ear Nose and Throat Symptom Survey in the COVID-19 Pandemic (CENTSS) among the Chinese population. This study aimed to assess the impact of COVID-19 infection on ear-related symptoms, with a specific focus on patients reporting tinnitus as a consequence of COVID-19 exposure. The findings from this study may contribute to improving management strategies for tinnitus in the context of long COVID. By enhancing our understanding of the factors contributing to tinnitus in individuals with COVID-19, health care providers can better tailor interventions to meet the specific needs of affected patients. Furthermore, this study serves as a foundation for future research on the long-term consequences of COVID-19 infection and associated tinnitus symptoms. Considering various risk factors for tinnitus, including infection, eustachian tube dysfunction, and hearing impairment [14], the outbreak of COVID-19, primarily a respiratory infection caused by the novel coronavirus SARS-CoV-2 [15], provides a substantial data set for analyzing the unique attributes of virus-induced tinnitus. Conducting extensive sample analyses would be highly beneficial in gaining comprehensive insights into the characteristics of individuals experiencing tinnitus after COVID-19 infection.

## Methods

### Study Design

A quantitative, online, cross-sectional survey study design was used to explore the impact of the COVID-19 pandemic on experiences with tinnitus in China. From December 19, 2022, to February 1, 2023, data were collected using wjx.cn, a widely used online questionnaire survey platform in China. The primary source of the samples in this study was from specific online platforms, namely “Guoke Patients,” and social media channels. To ensure sample diversity, the platform is characterized by a wide user base and a diverse user population. Simultaneously, social media channels were selected for their ability to reach user groups covering various ages, genders, professions, and educational backgrounds. Before completing the questionnaire, participants were required to read and confirm their voluntary agreement to participate. In the process of questionnaire data collection, we implemented a validation mechanism between questions to prevent duplicate submissions and the generation of false data. Furthermore, they could withdraw their participation at any time while completing the questionnaire. Only completely answered questionnaires were submitted. Each device could only access the questionnaire once, which is a restriction enforced by wjx.cn to ensure that each respondent only submits 1 questionnaire.

The inclusion criteria were as follows: (1) Chinese citizens currently residing in China, (2) age ≥18 years, (3) capable of independently reading and completing the self-administered questionnaire, and (4) willing to participate in the survey. Exclusion criteria included the following: (1) age younger than 18 years, (2) residing abroad, or (3) invalid data. All participants were anonymous adult volunteers. Thus, the survey posed no potential risk to participants’ physical and mental health. Furthermore, this study adhered to the ethical principles of the Measures for the Ethical Review of Biomedical Research Involving Humans of the National Health Commission of the People's Republic of China. 

### Ethical Considerations

Ethical approval for international data collection was obtained from the Ethics Panel at Fudan University’s Eye and ENT Hospital (number 2022127). This clinical study used a cross-sectional design, collecting only patient questionnaire information without intervening in the patients’ treatment plans or posing any physiological risks. The researchers were committed to safeguarding the confidentiality of the provided information and therefore requested exemption from obtaining informed consent. Patient questionnaire information was anonymized, and patient identities were not disclosed. All participants gave their informed consent online.

The CHERRIES (Checklist for Reporting Results of Internet E-Surveys) was used to report the methods and results of the survey (Multimedia Appendix 1).

Data were collected via an online survey from 1334 individuals in China, and after excluding responses with missing information, 1288 participants were included. The data for age, height, and weight showed significant variability, and the maximum values for these variables exceeded the means by 3 SDs. Therefore, using the median to describe the overall level may be more appropriate than the mean, which is heavily influenced by outliers.

### Survey Development

The design and implementation of the study and the data analysis were completed with the participation of 2 epidemiology professors. The survey captured the following categories: (1) demographic information such as name, age, height, weight, education, place of residence, and occupation (10 questions); (2) date of COVID-19 infection (1 question); (3) ear symptom–related questions (25 questions; eg, ear-related symptoms such as hearing loss, tinnitus, stuffy ears, ear pain, and dizziness; the impact of tinnitus as measured using the Tinnitus Handicap Inventory [3 questions] and the Athens Insomnia Scale [2 questions]; the level of impact on daily life due to ear symptoms after COVID-19 infection [5 questions]); (4) cognitive and emotional state after COVID-19 infection (6 questions); (5) past medical history of ear, nose, throat (ENT) diseases, hypertension, or diabetes (3 questions).

### Survey Distribution

Individuals who provided informed consent were considered eligible. Recruitment primarily took place through the social media channels of patient organizations, such as the Weixin and Guoke websites [16]. To participate in the survey, respondents were required to provide online informed consent, and the survey software restricted multiple submissions from the same IP address.

### Data Analysis

Initially, the data cleaning process involved the removal of cases that did not meet the eligibility criteria for the study. The data analysis used a mixed approach that included both quantitative and qualitative analyses. Descriptive statistics, such as frequencies, means, and SDs, were calculated using SPSSAU (QingSi Technology Ltd). If the data were normally distributed, they are expressed as the mean (SD), and Student *t* tests were used for comparisons between 2 groups. For the analysis of composition ratios, the chi-square test or Fisher exact probability method was used, as appropriate. For multivariate dichotomous dependent variable data, logistic regression analysis was used. Differences were considered statistically significant at *P*<.05. The chi-square test was used to examine the relationships between categorical variables, and adjusted residuals were used for post hoc analysis to determine significant relationships. To account for multiple testing, the *P* value was adjusted (Bonferroni) and set to be significant at *P*<.001. Qualitative data obtained from the open-ended questions were analyzed separately using inductive thematic analysis, and the identified themes were used to support the quantitative analysis. To explore the extent of the impact of the independent variables on the dependent variable, a logistic regression model was used to analyze the relationship between continuous variables, and a logistic regression model was used to handle categorical variables.

## Results

### Demographic Characteristics

A total of 1334 questionnaires were distributed to the registered users on the platform, of which 23 questionnaires were from foreigners, 23 were excluded due to being younger than 18 years, and 26 were considered otherwise invalid. The demographic characteristics of the study respondents are shown in Table 1.

**Table 1:** Demographic characteristics of the China Ear Nose and Throat Symptom Survey in the COVID-19 Pandemic (CENTSS 2023).

Variables		Tinnitus status			Total sample (n=1262)		*P* value	
		No (n=888)	Yes (n=374)					
Age (years), mean (SD)		37.26 (9.949)	36.265 (10.799)	36.965 (10.214)		.13		
**Gender, n (%)**								.05
	Female	688 (77.48)	308 (82.35)	996 (78.92)				
	Male	200 (22.52)	66 (17.65)	266 (21.08)				
**Age group (years)**								.03
	≤30	192 (21.62)	109 (29.14)	301 (23.85)				
	31-36	227 (25.56)	89 (23.80)	316 (25.04)				
	37-43	239 (26.91)	82 (21.93)	321 (25.44)				
	≥44	230 (25.90)	94 (25.13)	324 (25.67)				
Height (cm), mean (SD)		166.422 (50.672)	164.533 (7.168)	165.862 (42.678)		.47		
Weight (kg), mean (SD)		61.202 (12.070)	61.663 (11.980)	61.339 (12.040)		.53		
BMI (kg/m^2^), mean (SD)		22.420 (3.596)	22.685 (3.619)	22.499 (3.604)		.23		
**Education level, n (%)**								<.001
	Junior high school or less	32 (3.60)	33 (8.82)	65 (5.15)				
	Bachelor’s or associate degree	556 (62.61)	242 (64.71)	798 (63.23)				
	Master’s or higher degree	300 (33.78)	99 (26.47)	399 (31.62)				
**Occupation, n (%)**								.88
	Noise-exposed occupation	124 (13.96)	55 (14.71)	179 (14.18)				
	Non–noise-exposed occupation	540 (60.81)	222 (59.36)	762 (60.38)				
	Other occupation	224 (25.23)	97 (25.94)	321 (25.44)				
**Region in China, n (%)**								<.001
	East	712 (80.18)	250 (66.84)	962 (76.23)				
	Middle	99 (11.15)	69 (18.45)	168 (13.31)				
	West	77 (8.67)	55 (14.71)	132 (10.46)				
**ENT^a^ disease history, n (%)**								.003
	No	402 (45.27)	136 (36.36)	538 (42.63)				
	Yes	486 (54.73)	238 (63.64)	724 (57.37)				
**Hypertension, n (%)**								.89
	No	834 (93.92)	352 (94.12)	1186 (93.98)				
	Yes	54 (6.08)	22 (5.88)	76 (6.02)				
**Diabetes, n (%)**								.90
	No	879 (98.99)	361 (96.52)	1240 (98.25)				
	Yes	9 (1.01)	13 (3.48)	26 (1.74)				

^a^ENT: ear, nose, throat.

### Representation of Individuals With Tinnitus

**Figure 1:**
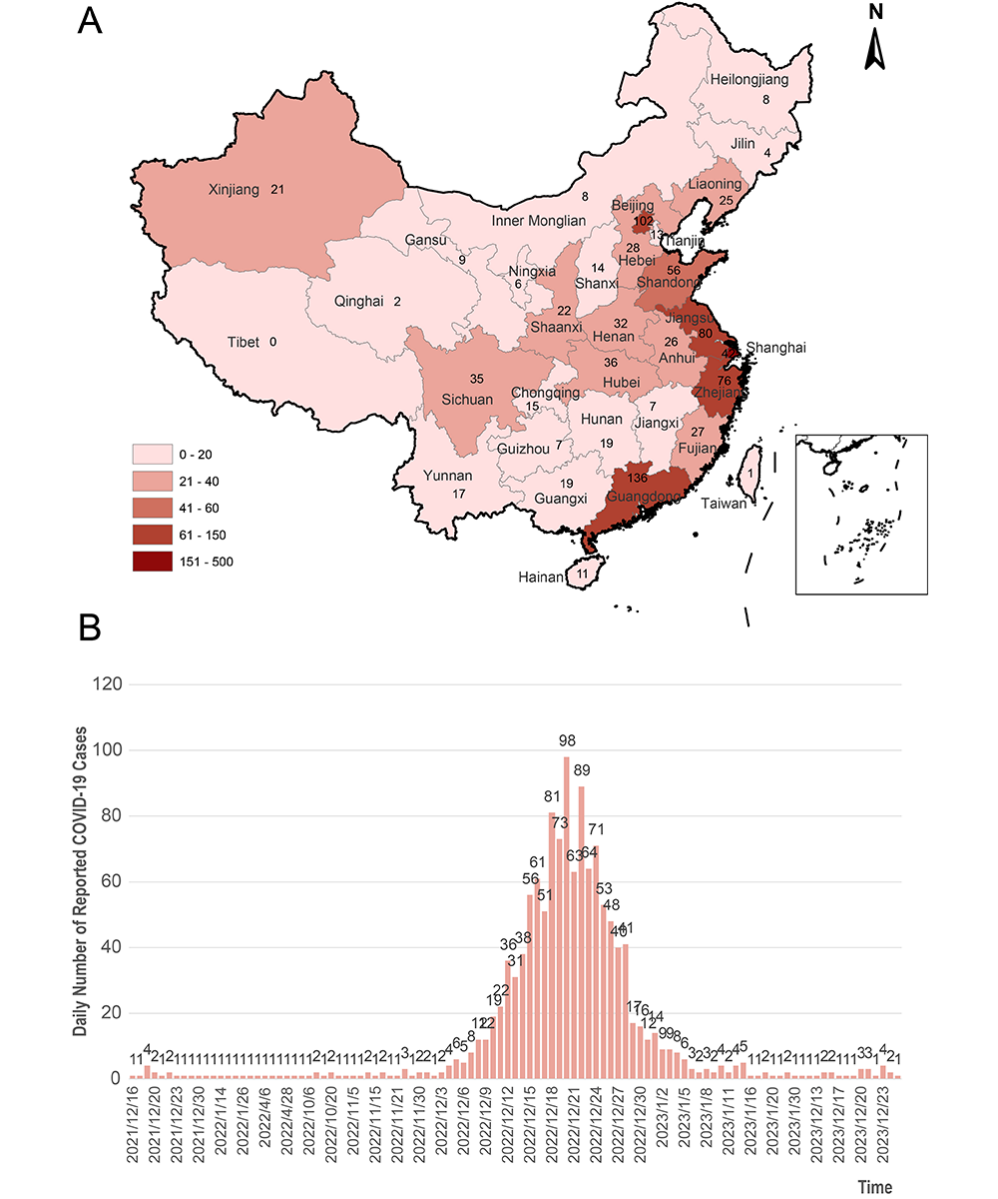
The spatiotemporal distribution of patients with COVID-19 who responded to the China Ear Nose and Throat Symptom Survey in the COVID-19 Pandemic (CENTSS 2023): (A) distribution and (B) timeline of COVID-19 infection.

After removing the 72 invalid responses, there were 1262 respondents aged from 18 years to 80 years representing 24 areas in China, although some areas were only marginally represented (Figure 1A).

### Timeline of COVID-19 Infection

As shown in Figure 1B, the survey respondents first reported having COVID-19 infection on December 16, 2021. The number of infected participants increased from early December 2022 to a peak on December 21, 2022, as policies were continuously adjusted. After the peak, the number of infected participants decreased and stabilized on January 20, 2023.

### Impact of the Pandemic on Ear Symptoms

**Figure 2:**
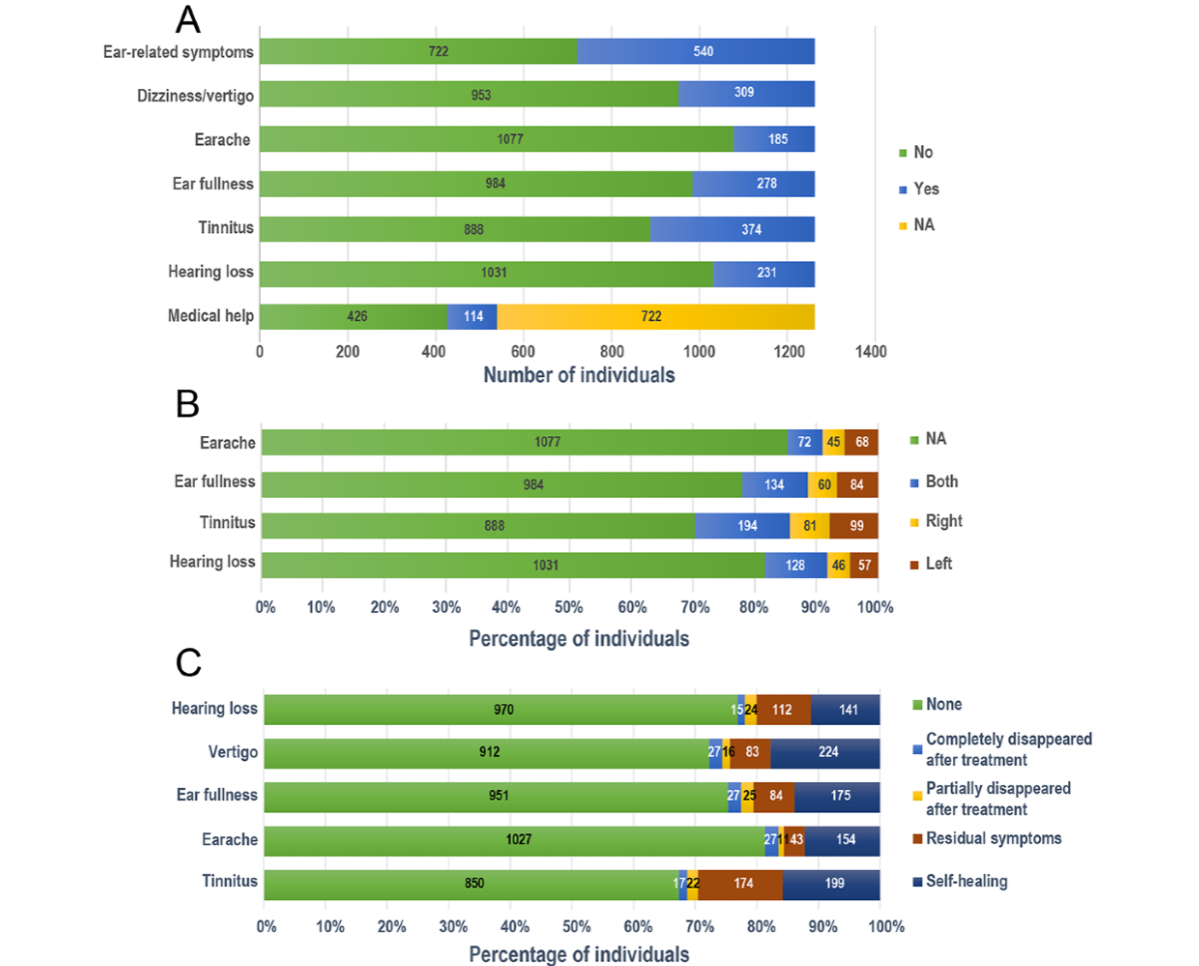
Based on responses to the China Ear Nose and Throat Symptom Survey in the COVID-19 Pandemic (CENTSS) cross-sectional study: (A) ear symptoms and visits to the doctor after COVID-19, (B) location of ear symptoms after COVID-19 infection, (C) outcome of ear symptoms after COVID-19 infection.

During the COVID-19 pandemic, 540 (42.79%) of the 1262 participants experienced some ear-related symptoms after being infected. Based on the self-reported symptoms of each of the 1262 participants, 374 (29.46%) had tinnitus, 185 (14.4%) had ear pain, 278 (21.74%) had ear fullness, 83 (17.99%) had hearing loss, and 309 (24.29%) experienced dizziness. A total of 114 (9%) of the 540 respondents with ear-related symptoms had seen a doctor because of their ear symptoms, but 426 (33.6%) of the 540 respondents did not go to the hospital (Figure 2A).

After COVID-19 infection, 556 (43.07%) of the 1262 respondents reported that they had ear-related problems, including earache, ear fullness, dizziness or vertigo, and hearing loss. Of the 185 participants experiencing ear pain, 72 (39%) of the respondents had pain in both ears. Of the 278 participants experiencing ear fullness, 134 (48.2%) experienced fullness in both ears. Of the 374 participants experiencing tinnitus, 194 (51.9%) experienced tinnitus in both ears. Of the 83 participants experiencing hearing loss, 128 (55.4%) experienced hearing loss in both ears (Figure 2B). Only a small number of the respondents treated their ear symptoms, and most of the patients healed on their own, but 174 patients still had residual tinnitus symptoms, 143 patients had residual earache, and 112 patients had residual hearing loss.

### Impact of Tinnitus After COVID-19 Infection

Because only 374 respondents had bothersome tinnitus, 888 respondents chose to skip the questions about the impact of tinnitus after COVID-19 infection. The respondents reported the impact of tinnitus on their quality of sleep, and they self-assessed the impact of ear symptoms, including tinnitus, earache, ear fullness, dizziness, and hearing loss, on their daily activities using a score ranging from 0 (no impact) to 10 (severe impact). Additionally, the study investigated the progression of ear-related symptoms in respondents who had contracted COVID-19. Table 2 shows that 146 (11.57%) of the 1262 respondents reported a slight impact on their sleep and that 117 (14.03%) were slightly dissatisfied with their quality of overall sleep. Tinnitus and vertigo had the greatest effects on respondents among all ear symptoms experienced after COVID-19 infection (Figure 2C).

**Table 2:** The impact of tinnitus on sleep and ear symptom score after COVID-19 infection according to the China Ear Nose and Throat Symptom Survey in the COVID-19 Pandemic (CENTSS 2023; n=1262).

Questions			Results
**Did the tinnitus affect your sleep? (eg, difficulty falling asleep, waking up at night, early awakening), n (%)**			
	No effect	163 (12.92)	
	Slight impact	146 (11.57)	
	Significant impact	54 (4.28)	
	Severe impact or no sleep	11 (0.87)	
**Quality of overall sleep, n (%)**			
	Satisfied	73 (5.78)	
	Slightly dissatisfied	177 (14.03)	
	Significantly dissatisfied	111 (8.08)	
	Very dissatisfied or couldn't sleep	13 (1.03)	
**Level of impact on daily life due to ear symptoms after COVID-19 infection, mean (SD)**			
	Tinnitus	1.366 (2.509)	
	Earache	0.692 (1.765)	
	Stuffy ear	1.062 (2.267)	
	Vertigo	1.193 (2.310)	
	Hearing loss	0.956 (2.129)	

### Cognitive and Emotional State After COVID-19 Infection

**Figure 3:**
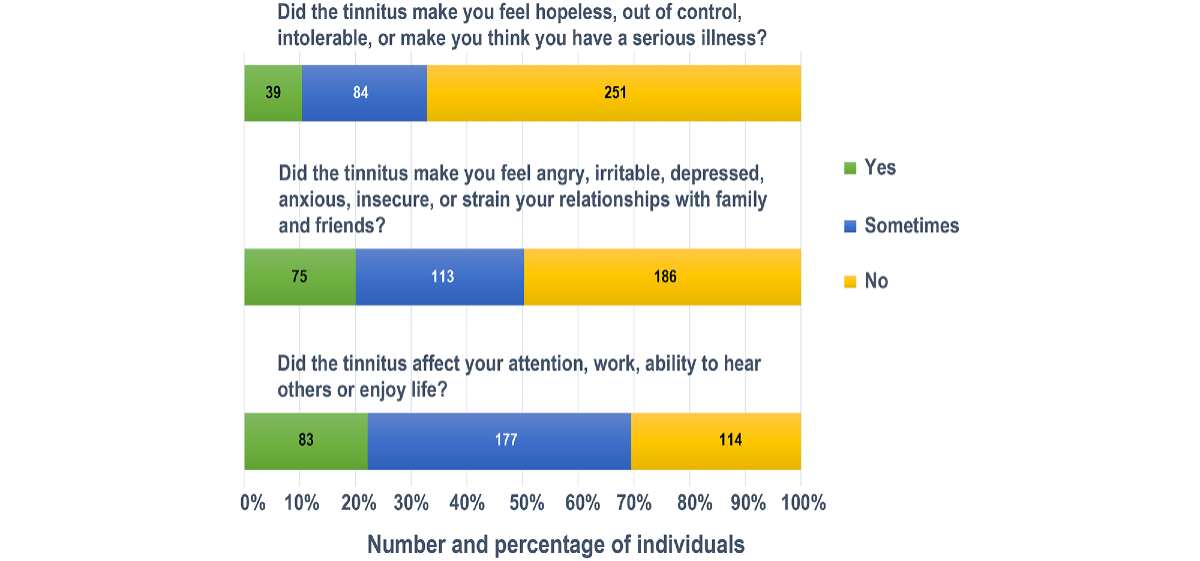
Cognitive and emotional impact of tinnitus for respondents of the China Ear Nose and Throat Symptom Survey in the COVID-19 Pandemic (CENTSS) cross-sectional study (2023).

People had different cognitive and emotional states after COVID-19 infection. Of the 888 participants with no tinnitus, 281 (31.6%) said they were able to remember most things but had some difficulty thinking and solving daily problems, while 137 (36.63%) of the participants with tinnitus reported the same. Of the 374 participants with tinnitus, 60 (16%) said they were somewhat forgetful but clear-minded and able to solve daily problems, while only 120 (13.5%) of the 888 participants without tinnitus reported the same. Most of the participants with tinnitus (163/374, 43.6%) said they were somewhat unhappy, while only 28.7% (255/888) of the participants without tinnitus reported being unhappy (Figure 3). Table 3 shows the results of the cognitive and emotional state after COVID-19 infection. These findings showed that experiencing tinnitus after COVID-19 infection was associated with a worse cognitive and emotional state.

**Table 3:** Cognitive and emotional state of the respondents of the China Ear Nose and Throat Symptom Survey in the COVID-19 Pandemic (CENTSS 2023).

Cognitive and emotional state after COVID-19 infection		Tinnitus status, n (%)			Total sample (n=1262), n (%)		χ^2^ (*df*)			*P* value	
		No (n=888)	Yes (n=374)								
**Cognitive state after COVID-19 infection**								51.661 (10)			<.001
	Able to remember most things, be clear-minded, and solve daily problems	0	1 (0.27)	1 (0.08)							
	Able to remember most things but have some difficulty thinking and solving daily problems	281 (31.64)	137 (36.63)	418 (33.12)							
	Somewhat forgetful but clear-minded and able to solve daily problems	74 (8.33)	60 (16.04)	134 (10.62)							
	Somewhat forgetful and have some difficulty thinking and solving daily problems	120 (13.51)	61 (16.31)	181 (14.34)							
	Very forgetful and have great difficulty thinking and solving daily problems	407 (45.83)	104 (27.81)	511 (40.49)							
	Unable to remember anything and unable to think and solve daily problems	6 (0.68)	11 (2.94)	17 (1.35)							
**Emotional state after COVID-19 infection**								45.116 (8)			<.001
	So unhappy to the point of feeling life has no value	21 (2.36)	19 (5.08)	40 (3.17)							
	Happy and interested in life	323 (36.37)	86 (22.99)	409 (32.41)							
	Somewhat unhappy	255 (28.72)	163 (43.58)	418 (33.12)							
	Somewhat happy	252 (28.38)	82 (21.93)	334 (26.47)							
	Very unhappy	37 (4.17)	24 (6.42)	61 (4.83)							
**In the past two weeks, how often have you felt nervous, anxious, or restless?**								58.004 (6)			<.001
	More than half of the days	123 (13.85)	99 (26.47)	222 (17.59)							
	Almost every day	45 (5.07)	39 (10.43)	84 (6.66)							
	A few days	454 (51.13)	179 (47.86)	633 (50.16)							
	Not at all	266 (29.95)	57 (15.24)	323 (25.59)							
**In the past two weeks, how often have you been unable to stop or control worrying?**								71.09 (6)			<.001
	More than half of the days	82 (9.23)	82 (21.93)	164 (13)							
	Almost every day	37 (4.17)	40 (10.70)	77 (6.1)							
	A few days	383 (43.13)	153 (40.91)	536 (42.47)							
	Not at all	386 (43.47)	99 (26.47)	485 (38.43)							
**In the past two weeks, how often have you felt a lack of energy or interest in doing things?**								46.517 (6)			<.001
	More than half of the days	171 (19.26)	101 (27.01)	272 (21.55)							
	Almost every day	76 (8.56)	67 (17.91)	143 (11.33)							
	A few days	399 (44.93)	151 (40.37)	550 (43.58)							
	Not at all	242 (27.25)	55 (14.71)	297 (23.53)							
**In the past two weeks, how often have you felt down, depressed, or hopeless?**								45.511 (6)			<.001
	More than half of the days	101 (11.37)	81 (21.66)	182 (14.42)							
	Almost every day	41 (4.62)	28 (7.49)	69 (5.47)							
	A few days	398 (44.82)	181 (48.40)	579 (45.88)							
	Not at all	348 (39.19)	84 (22.46)	432 (34.23)							

## Discussion

### Principal Findings: The Incidence of Tinnitus Increased After the COVID-19 Pandemic

Overall, tinnitus was rated as more bothersome during the pandemic than before. During the COVID-19 pandemic, like many case reports, the onset or aggravation of tinnitus was documented [17-20]. Of the 1262 participants, 540 (42.79%) reported experiencing some ear-related symptoms after being infected, and the most common ear-related symptoms were vertigo and tinnitus. Despite this, only 114 (9.03%) of the participants had seen a doctor regarding their ear symptoms, and 426 (33.75%) did not visit the hospital. COVID-19 infection may present with otological manifestations such as hearing loss, tinnitus, vertigo, ear fullness, and earache [18]. Even if some patients experienced ear symptoms, these were mostly transitory, although some studies have shown evidence for the worsening of preexisting tinnitus as a possible long-term effect of COVID-19 [21,22]. In our practice, we administer questionnaires to patients to assess how troublesome the tinnitus is for them, as well as how it affects their daily activity and ability to function. Most of the surveyed participants have experienced the distress caused by tinnitus, including its impact on attention, emotions, anxiety, depression, and sleep quality. Increased levels of anxiety, depression, and irritability have been reported [23]. These emotional factors are associated with exacerbated tinnitus annoyance. Negative emotions were made worse by a variety of worries and frustrations, such as relationship issues that increased because of confinement, concerns about the availability of food, and worries about contracting the virus. Due to loss of employment, furloughs, and a decrease in the value of investment, financial worries have also caused tinnitus to become significantly worse. These results are consistent with a recent systematic review on the effects of COVD-19 on mental health [24], which found reduced psychological well-being and increased levels of anxiety and depression in the general population compared with before COVID-19. However, less than 10% of individuals with tinnitus sought medical attention after COVID-19 infection, and the management and provision of health care for subsequent individuals with tinnitus need attention. Nocini et al [25] reported that the worldwide burden of tinnitus may have increased significantly during the ongoing COVID-19 pandemic. To improve tinnitus care, better awareness and more accessible resources and management are crucial. Tracking and managing hearing-related changes due to having COVID-19 will be important as clinical services resume.

COVID-19 is an infectious respiratory disease caused by severe acute respiratory syndrome and other systematic symptoms [26]. In some cases, the tinnitus resolved on its own after the resolution of the COVID-19 infection, while in other cases, it persisted even after recovery from the virus. In order to understand the prevalence of tinnitus and ear symptoms after COVID-19 infection, we conducted an epidemiological questionnaire-based survey at the end of 2022 and used regression analysis to identify factors affecting patients' tinnitus. Table 4 shows the association of gender, age group, height, weight, BMI, education level, occupation, region in China, ENT disease history, hypertension history, and diabetes history in relation to tinnitus after COVID-19, and gender, age group, education level, region in China, and ENT disease history were added into the model. The regression model likelihood ratio test resulted in χ^2^_5_=39.310 and *P*<.001. For the Hosmer–Lemeshow test, the results were χ^2^_8_=5.362 and *P*=.72. The regression model was ln(p/1-p)=–1.364 – (0.211*gender) – (0.104*age group) – (0.106*education level) + (0.416*region in China) + (0.387*ENT medical history).

**Table 4:** Regression analysis results of the China Ear Nose and Throat Symptom Survey in the COVID-19 Pandemic (CENTSS 2023).

Variables			Univariable analysis				Multivariable analysis		
			OR^a^ (95% CI)		*P* value		OR (95% CI)		*P* value
**Gender**					.05				.10
	Female	Reference				Reference			
	Male	0.737 (0.541-1.004)				0.762 (0.552-1.051)			
**Age group (years)**					.04				
	≤30	Reference				Reference		—^b^	
	31-36	0.691 (0.492- 0.970)				0.682 (0.482-0.966)		.03	
	37-43	0.604 (0.429- 0.852)				0.599 (0.421-0.852)		.004	
	≥44	0.72 (0.515-1.007)				0.698 (0.492-0.991)		.04	
Height			0.996 (0.981-1.011)		.61		—		—
Weight			1.003 (0.993-1.013)		.53		—		—
BMI			1.02 (0.987-1.055)		.23		—		—
**Education level**					.10				
	Junior high school (or below)	Reference				Reference		—	
	Bachelor’s (or associate) degrees	0.422 (0.254-0.702)				0.401 (0.236-0.682)		<.001	
	Master’s (or above) degrees	0.32 (0.187-0.547)				0.308 (0.176-0.538)		<.001	
**Occupation**					.99				
	Noise exposure occupation	Reference				—		—	
	Non noise exposure occupation	0.927 (0.651-1.320)				—		—	
	Other occupation	0.976 (0.656-1.452)				—		—	
**Region in China**					<.001				
	East	Reference				Reference		—	
	Middle	1.985 (1.414-2.787)				1.891 (1.336-2.678)		<.001	
	West	2.034 (1.398-2.959)				2.063 (1.408-3.022)		<.001	
**ENT^c^ disease history**					.04				*<*.001
	No	Reference				Reference			
	Yes	1.448 (1.129-1.856)				1.530 (1.184-1.977)			
**Hypertension**					.89				—
	No	Reference				—			
	Yes	0.965 (0.579-1.609)				—			
**Diabetes**					.90				—
	No	Reference				—			
	Yes	1.055 (0.455-2.448)				—			

^a^OR: odds ratio.

^b^Not applicable.

^c^ENT: ear, nose, throat.

In our survey, female participants, younger participants (<30 years), less educated participants, participants from western China, and participants with a history of otolaryngology diseases were more likely to develop tinnitus after COVID-19 infection. There were 688 (688/888, 77.8%) female participants compared with 200 male participants, and this may have caused selective sex-related bias. Interestingly, this conclusion differs from the known risk factors for tinnitus before COVID-19 infection, such as long-term noise exposure, ototoxic drugs, aging, and genetic predispositions, and concomitant neural alterations are considered the initial source of tinnitus. There is also no unified conclusion on the relationship between tinnitus and gender. Several studies have reported a higher incidence of tinnitus among men than women [27] and that women, especially menopausal women, are more susceptible to tinnitus and developing annoying tinnitus [27,28]. Similar studies confirmed that women and younger individuals are more likely to complain about tinnitus [29]. Studies on COVID-19 revealed that ENT symptoms are observed more frequently in younger age groups and in women [30-32]. From the explanations provided, it appears that the unified conclusion on the relationship between tinnitus and gender may be partly attributed to significant lifestyle changes in these groups during the pandemic. These include changes in employment, increased childcare, and household responsibilities. A study involving patients with tinnitus at a clinic in Germany found that COVID-19 increased grief, frustration, stress, and nervousness, although there was only a small increase in tinnitus distress compared with 2 years prior to lockdown [31]. As documented in previous studies [33], the COVID-19 pandemic is a potential environmental stressor that might influence individually perceived tinnitus distress. Because not all people have been affected by the pandemic in the same way, the situation allows one to identify environmental factors and personality traits that impact tinnitus distress differently. The risk factors for tinnitus among respondents with a history of ENT problems were 1.53 times higher than in patients without a history of ENT problems, similar to the conclusions of the study by Mui et al [18] involving 365 older adult respondents in Australia and other countries where tinnitus was reported to be more bothersome during the pandemic, by 36% of respondents, whereas 59% reported no change and 5% reported less bothersome tinnitus. As for the regional distribution, people in the western region of China were 2.063 times more likely to report tinnitus compared with those in the eastern region, and the odds ratio in the central region was 1.891 compared with people in the eastern region. This may be because of economic and environmental factors.

Although it is still not entirely clear why some patients with COVID-19 develop tinnitus, it is speculated that this may be related to inflammation or other changes in the body as a result of the virus [34]. Regarding the possible etiologies of tinnitus during the COVID-19 pandemic, one plausible explanation may be attributed to direct viral injury to the ear. However, the elevated levels of anxiety and stress experienced by individuals during this global health crisis have been identified as exacerbating factors that may have significantly contributed to the worsening or propagation of this debilitating condition [35]. Therefore, the increased burden of anxiety and stress during the pandemic may have played a significant role in the worsening of tinnitus symptoms. More research is needed to explore the relationship between tinnitus and the psychosocial impact of the COVID-19 pandemic. Similar to previous research [32,36,37], we also found that tinnitus was more likely to occur in participants in the western region in China and in participants with low education levels. Differences in tinnitus onset between high- and low-income groups may be because the pressure of work and unemployment caused by COVID-19 infection was more readily reflected in people from the western region and in people with low education levels.

To highlight the impact of COVID-19 on the ear, our focus was on tinnitus along with other ear-related symptoms. COVID-19 infection can potentially lead to symptoms such as ear fullness, ear pain, vertigo, hearing loss, and even deafness. Ear fullness and ear pain may arise from the virus invading the external auditory canal, eustachian tube, or nerves innervating the outer ear. Vertigo may be caused by viral invasion of the inner ear, affecting both the vestibule and cochlea or the vestibulocochlear nerve. Hearing loss or deafness can result from the virus damaging the cochlear hair cells while sparing the vestibular system. It is important to note that different symptoms may indicate viral invasion of different parts of the ear, and this should be considered during diagnosis and treatment. Physicians must remain vigilant and take measures to minimize the detrimental effects of COVID-19 in order to protect the patients' auditory function to the greatest extent possible.

On a worldwide level [38-40], lower health development index and total health expenditure or gross domestic product per capita have been associated with a higher prevalence of moderate to severe ear symptoms.

### Limitations and Future Directions

There are some limitations in the interpretation of this study that should be considered. First, our study has the limitations of a cross-sectional survey such as convenience sampling and possible recall bias. Second, this online survey potentially introduced selection bias because participants were limited to only those who could access the internet. Therefore, the generalizability of the findings needs to be interpreted with caution. Future studies should include longitudinal follow-up periods to identify the trajectory of the symptoms in order to indicate whether the tinnitus resolves or remains and if the severity changes.

### Conclusion

This study found that tinnitus has been reported to be more distressing during the pandemic than before it, and individuals experiencing tinnitus after a COVID-19 infection were found to have poorer cognitive and emotional well-being. The presence of various ear-related symptoms in patients following a COVID-19 infection may indicate viral involvement in different parts of the ear. Therefore, it is crucial to monitor and address any changes in hearing associated with COVID-19 as clinical services resume.

## Appendix

Multimedia Appendix 1 CHERRIES (Checklist for Reporting Results of Internet E-Surveys).

## Abbreviations

CENTSS: China Ear Nose and Throat Symptom Survey in the COVID-19 Pandemic
CHERRIES: Checklist for Reporting Results of Internet E-Surveys
ENT: ear, nose, throat
NICE: National Institute for Health and Care Excellence

## Data Availability

The data sets generated or analyzed during this study are available from the corresponding author upon reasonable request.
