# Supplementary material for: Exploring the Prevalence of Tinnitus and Ear-Related Symptoms in China After the COVID-19 Pandemic: Online Cross-Sectional Survey
Source: JMIR Form Res. 2024 Apr 24;8:e54326. doi: 10.2196/54326 (PMC11045005; doi:10.2196/54326)
Supplement: Multimedia Appendix 1 [file formative_v8i1e54326_app1.pdf]

Table 1. Checklist for Reporting Results of Internet E-Surveys (CHERRIES)

Item Category	Checklist Item	Page Number	Description
**Design**			
	Study design	4	It is an online cross-sectional survey.<br>Study population are Chinese citizens currently residing in China, age ≥18 years, apable of independently reading and completing the self-administered questionnaire, and willing to participate in the survey.
**IRB (Institutional Review Board) approval and informed consent process**			
	IRB approval	5	Ethical approval for international data collection was obtained from the Ethics Panel at Fudan University's Eye and ENT Hospital (number 2022127).
	Informed consent	5	All participates gave their informed consent online.
	Data protection	5	Patient questionnaire information was anonymized , and patient identities were not disclosed.
**Development and pre-testing**			
	Development and testing	5	The survey captured the following categories:<br>(1) demographic information such as name, age, height, weight, education, place of residence, and occupation (10 questions);<br>(2) date of COVID-19 infection (1 question);<br>(3) ear symptom–related questions (25 questions);<br>(4) cognitive and emotional state after COVID-19 infection (6 questions);<br>(5) past medical history of ear, nose, throat (ENT) diseases, hypertension, or diabetes (3 questions).<br>Questionnaire was developed and reviewed by research team and then piloted before distribution.
**Recruitment process and description of the sample having access to the questionnaire**			
	Open survey	4	The data of our study were collected using wjx.cn, a widely used online questionnaire survey platform in China.
	Contact and Advertising	4	The primary source of the samples in this study was from specific online platforms, namely "Guoke Patients" and social media channels.
**Survey administration**			
	Web	4	It was posted on a Website "Guoke patients"<br>https://mp.weixin.qq.com/s/89XRg6KUqVXQ0dGOfFDwng
			Since the outbreak of the novel coronavirus, systemic symptoms following COVID-19 infection have been frequently observed. Among them, nasal congestion, sore throat, cough, and other otolaryngological symptoms are common early symptoms of COVID-19 infection. Additionally, symptoms such as tinnitus and loss of smell have also been widely reported.

Table 1. Checklist for Reporting Results of Internet E-Surveys (CHERRIES)

Item Category	Checklist Item	Page Number	Description
	Context	The context was written in the survey questionnaire.	In order to investigate otolaryngological symptoms following COVID-19 infection, the Otorhinolaryngology Hospital affiliated with Fudan University designed this questionnaire. The questionnaire includes a series of otolaryngological symptoms following COVID-19 and their progression, inviting COVID-19 patients from both domestic and international regions to fill it out. Each questionnaire greatly contributes to subsequent research. Your questionnaire will be eligible to receive symptom guidance and follow-up contact from the professional team at the Otorhinolaryngology Hospital affiliated with Fudan University. Please scan the QR code or click the link to fill out the questionnaire. Thank you for your support!
	Voluntary	5	It was a voluntary survey to be filled in by every visitor who wanted to enter the Website.
	Incentives	The context was written in the survey questionnaire.	No incentives were offered in the survey.
	Time/Date	5	It was conducted from December 19, 2022, to February 1, 2023.
	Number of Items	The context was written in the survey questionnaire.	The survey has 45 questions.
	Completeness check	The context was written in the survey questionnaire.	The system conducts a completeness check before the questionnaire is submitted, and it is impossible to submit without completing mandatory questions.
**Preventing multiple entries from the same individual**	*Unique site visitor*	The context was written in the survey questionnaire.	To avoid duplicate submissions, each survey can only be filled out once per IP address.
**Analysis**	*Statistical analysis and data handling*	6	Exclusive inclusion was granted solely to questionnaires demonstrating comprehensive responses within the dataset. Calculations encompassed the identification of information sources and their respective frequencies of utilization, the evaluation of social media engagement alongside its frequency, and the derivation of well-being scores. Furthermore, regression analysis was conducted to explore pertinent relationships.

Notes: Adapted from: Eysenbach G. Improving the quality of Web surveys: the Checklist for Reporting Results of Internet E-Surveys (CHERRIES). J Med Internet Res. 2004;6(3):e34. doi: 10.2196/jmir.6.3.e34. PMID: 15471760. PMCID: PMC1550605. Available from: https://www.jmir.org/2004/3/e34/.

© Gunther Eysenbach. Originally published in the Journal of Medical Internet Research (http://www.jmir.org), 29.9.2004. Creative Commons Attribution License (http://www.creativecommons.org/licenses/by/2.0/).
